# (5*Z*,7*Z*)-6,8-Dimethyl-9*H*-tetra­zolo[1,5-*b*][1,2,4]triazepine

**DOI:** 10.1107/S160053680904392X

**Published:** 2009-10-28

**Authors:** Chun-Lin He, Zhi-Ming Du, Zheng-Qiang Tang, Xiao-Min Cong, Ling-Qiao Meng

**Affiliations:** a State Key Laboratory of Explosion Science and Technology, Beijing Institute of Technology, Beijing 100081, People’s Republic of China

## Abstract

The mol­ecule of the title compound, C_6_H_8_N_6_, is approximately planar, with a maximum deviation from planarity of 0.099 (1) Å. In the crystal, mol­ecules are linked to each other *via* pairs of N—H⋯N hydrogen bonding, forming inversion dimers. The crystal structure is further stabilized by π–π stacking inter­actions, with a centroid–centroid distance of 3.419 (1) Å.

## Related literature

For the preparation of the title compound, see: Gaponnik & Karavai (1984 ▶). For applications of fused tetra­zole ring compounds, see: Taha 2007 ▶; Zbigniew *et al.* (2007 ▶); Galvez-Ruiz *et al.* (2005 ▶); Klapötke & Sabaté (2008 ▶). For related structures, see: Taha (2005 ▶); He *et al.* (2009*a* ▶,*b* ▶).
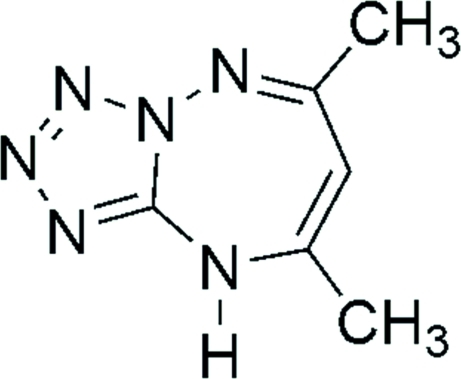


## Experimental

### 

#### Crystal data

C_6_H_8_N_6_
*M* *_r_* = 164.18
Monoclinic, 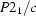

*a* = 3.9184 (8) Å
*b* = 13.584 (3) Å
*c* = 13.767 (3) Å
β = 96.274 (3)°
*V* = 728.4 (3) Å^3^
*Z* = 4
Mo *K*α radiation
μ = 0.11 mm^−1^
*T* = 93 K
0.47 × 0.33 × 0.13 mm

#### Data collection

Rigaku Saturn 724+ diffractometer
Absorption correction: none
5029 measured reflections
1647 independent reflections
1445 reflections with *I* > 2σ(*I*)
*R*_int_ = 0.019

#### Refinement

*R*[*F* ^2^ > 2σ(*F* ^2^)] = 0.036
*wR*(*F* ^2^) = 0.082
*S* = 1.00
1647 reflections
119 parameters
H atoms treated by a mixture of independent and constrained refinement
Δρ_max_ = 0.26 e Å^−3^
Δρ_min_ = −0.22 e Å^−3^

### 

Data collection: *CrystalClear* (Rigaku, 2008 ▶); cell refinement: *CrystalClear*; data reduction: *CrystalClear* ; program(s) used to solve structure: *SHELXS97* (Sheldrick, 2008 ▶); program(s) used to refine structure: *SHELXL97* (Sheldrick, 2008 ▶); molecular graphics: *ORTEP-3* (Farrugia, 1997 ▶); software used to prepare material for publication: *WinGX* (Farrugia, 1999 ▶).

## Supplementary Material

Crystal structure: contains datablocks I, global. DOI: 10.1107/S160053680904392X/wn2354sup1.cif

Structure factors: contains datablocks I. DOI: 10.1107/S160053680904392X/wn2354Isup2.hkl

Additional supplementary materials:  crystallographic information; 3D view; checkCIF report

## Figures and Tables

**Table 1:** Hydrogen-bond geometry (Å, °)

*D*—H⋯*A*	*D*—H	H⋯*A*	*D*⋯*A*	*D*—H⋯*A*
N5—H5*N*⋯N4^i^	0.920 (19)	1.999 (19)	2.9156 (17)	173.5 (15)

Symmetry code: (i) 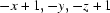.

**Fig. 1.**
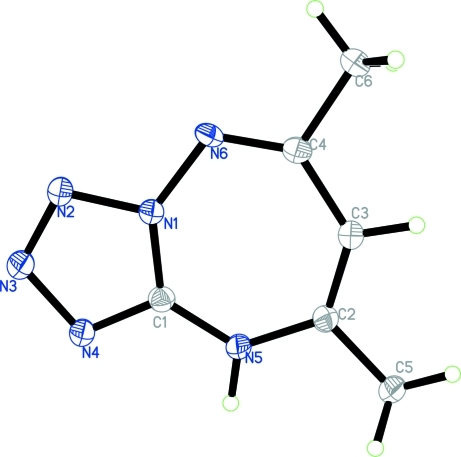
The molecular structure of the title compound with the atom-numbering scheme. Displacement ellipsoids are drawn at the 50% probability level. H atoms are represented as spheres of arbitrary radius.

**Fig. 2.**
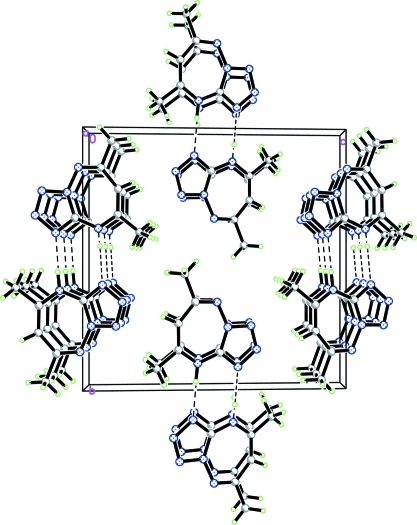
The packing of the title compound, viewed along the a-axis, showing the formation of dimers via N—H···N hydrogen bonds (dashed lines).

## supplementary crystallographic information

### Comment

Compounds based on the tetrazole ring have generated much interest (Taha 2005). On the one hand, fused tetrazole derivatives play an important role in many biological activities (Taha 2007; Zbigniew *et al.*,2007); on the other hand, nitrogen-rich compounds, in particular those containing the tetrazole ring, have great potential for energetic applications (Klapötke & Sabaté 2008); Galvez-Ruiz *et al.*2005). The title compound was first prepared by Gaponnik & Karavai (1984), but its crystal structure has hitherto not been reported. Here, we present the crystal structure of the title compound.

The molecular structure is shown in Fig.1. The molecule of the title compound assumes an approximately planar structure. The maximum deviation from planarity is 0.099 (1) Å for atom C3. Bond distances and angles are similar to the corresponding distances and angles reported for related compounds (He *et al.*2009*a*; He *et al.*2009*b*).

In the crystal structure, molecules are linked to each other *via* N—H···N hydrogen bonding (Table 1), forming a dimer structure. Intermolecular π-π interactions with a centroid···centroid distance of 3.419 (1) Å (Table 2), further help to stabilize the crystal structure. The crystal packing of the title compound is shown in Fig. 2, viewed down the *a* axis.

### Experimental

The title compound was obtained according to the literature method (Gaponnik & Karavai, 1984). The purity of the compound was checked by determining its melting point, 477–479 K. Single crystals suitable for X-ray crystal structure determination were obtained by slow evaporation of an acetone solution at room temperature over two days.

### Refinement

The H atom directly attached to the triazepine ring and that bonded to N were located in difference Fourier maps and refined freely. Methyl H atoms were placed in calculated positions, with C—H = 0.98 Å and refined as riding; *U*_iso_(H) = 1.2*U*_eq_(C).

### Crystal data

**Table:**

C_6_H_8_N_6_	*F*(000) = 344
*M**_r_* = 164.18	*D*_x_ = 1.497 Mg m^−^^3^
Monoclinic, *P*2_1_/*c*	Melting point: 477 K
Hall symbol: -P 2ybc	Mo *K*α radiation, λ = 0.71073 Å
*a* = 3.9184 (8) Å	Cell parameters from 2263 reflections
*b* = 13.584 (3) Å	θ = 3.3–27.5°
*c* = 13.767 (3) Å	µ = 0.11 mm^−^^1^
β = 96.274 (3)°	*T* = 93 K
*V* = 728.4 (3) Å^3^	Chunk, red
*Z* = 4	0.47 × 0.33 × 0.13 mm

### Data collection

**Table:**

Rigaku Saturn 724+ diffractometer	1445 reflections with *I* > 2σ(*I*)
Radiation source: Rotating Anode	*R*_int_ = 0.019
graphite	θ_max_ = 27.5°, θ_min_ = 3.3°
Detector resolution: 28.5714 pixels mm^-1^	*h* = −4→5
multi–scan	*k* = −17→17
5029 measured reflections	*l* = −11→17
1647 independent reflections	

### Refinement

**Table:**

Refinement on *F*^2^	Primary atom site location: structure-invariant direct methods
Least-squares matrix: full	Secondary atom site location: difference Fourier map
*R*[*F*^2^ > 2σ(*F*^2^)] = 0.036	Hydrogen site location: inferred from neighbouring sites
*wR*(*F*^2^) = 0.082	H atoms treated by a mixture of independent and constrained refinement
*S* = 1.00	*w* = 1/[σ^2^(*F*_o_^2^) + (0.031*P*)^2^ + 0.36*P*] where *P* = (*F*_o_^2^ + 2*F*_c_^2^)/3
1647 reflections	(Δ/σ)_max_ < 0.001
119 parameters	Δρ_max_ = 0.26 e Å^−^^3^
0 restraints	Δρ_min_ = −0.22 e Å^−^^3^

### Special details

**Table:**

Geometry. All e.s.d.'s (except the e.s.d. in the dihedral angle between two l.s. planes) are estimated using the full covariance matrix. The cell e.s.d.'s are taken into account individually in the estimation of e.s.d.'s in distances, angles and torsion angles; correlations between e.s.d.'s in cell parameters are only used when they are defined by crystal symmetry. An approximate (isotropic) treatment of cell e.s.d.'s is used for estimating e.s.d.'s involving l.s. planes.
Refinement. Refinement of *F*^2^ against ALL reflections. The weighted *R*-factor *wR* and goodness of fit *S* are based on *F*^2^, conventional *R*-factors *R* are based on *F*, with *F* set to zero for negative *F*^2^. The threshold expression of *F*^2^ > σ(*F*^2^) is used only for calculating *R*-factors(gt) *etc*. and is not relevant to the choice of reflections for refinement. *R*-factors based on *F*^2^ are statistically about twice as large as those based on *F*, and *R*- factors based on ALL data will be even larger.

### Fractional atomic coordinates and isotropic or equivalent isotropic displacement parameters (Å^2^)

**Table:**

	*x*	*y*	*z*	*U*_iso_*/*U*_eq_	
N1	0.4467 (3)	0.24707 (7)	0.45448 (7)	0.0165 (2)	
N2	0.5870 (3)	0.24983 (8)	0.36816 (7)	0.0208 (2)	
N3	0.6547 (3)	0.16030 (8)	0.34635 (8)	0.0223 (3)	
N4	0.5650 (3)	0.09674 (8)	0.41698 (7)	0.0195 (2)	
N5	0.3238 (3)	0.11503 (8)	0.56546 (8)	0.0207 (3)	
N6	0.3868 (3)	0.34046 (7)	0.49433 (7)	0.0176 (2)	
C1	0.4389 (3)	0.15238 (9)	0.48318 (8)	0.0163 (3)	
C2	0.1660 (3)	0.16746 (9)	0.63408 (8)	0.0165 (3)	
C3	0.1175 (3)	0.26544 (9)	0.63462 (9)	0.0180 (3)	
C4	0.2395 (3)	0.34313 (9)	0.57359 (9)	0.0165 (3)	
C5	0.0598 (3)	0.10241 (9)	0.71313 (9)	0.0202 (3)	
H5A	0.2595	0.0819	0.7548	0.024*	
H5B	−0.0569	0.0456	0.6844	0.024*	
H5C	−0.0914	0.1380	0.7509	0.024*	
C6	0.1945 (3)	0.44529 (9)	0.61191 (9)	0.0206 (3)	
H6A	0.2920	0.4920	0.5705	0.025*	
H6B	0.3084	0.4503	0.6771	0.025*	
H6C	−0.0457	0.4588	0.6126	0.025*	
H3	0.006 (4)	0.2905 (11)	0.6876 (11)	0.025 (4)*	
H5N	0.342 (4)	0.0478 (14)	0.5724 (12)	0.036 (5)*	

### Atomic displacement parameters (Å^2^)

**Table:**

	*U*^11^	*U*^22^	*U*^33^	*U*^12^	*U*^13^	*U*^23^
N1	0.0214 (5)	0.0138 (5)	0.0148 (5)	0.0006 (4)	0.0043 (4)	0.0002 (4)
N2	0.0285 (6)	0.0182 (5)	0.0167 (5)	0.0010 (4)	0.0074 (4)	0.0004 (4)
N3	0.0309 (6)	0.0180 (5)	0.0191 (5)	0.0013 (5)	0.0078 (4)	0.0014 (4)
N4	0.0261 (6)	0.0167 (5)	0.0166 (5)	0.0007 (4)	0.0062 (4)	0.0005 (4)
N5	0.0315 (6)	0.0130 (5)	0.0193 (5)	0.0016 (4)	0.0106 (5)	0.0019 (4)
N6	0.0230 (5)	0.0110 (5)	0.0189 (5)	0.0009 (4)	0.0030 (4)	−0.0018 (4)
C1	0.0177 (6)	0.0151 (6)	0.0161 (6)	0.0000 (5)	0.0013 (4)	0.0005 (4)
C2	0.0174 (6)	0.0168 (6)	0.0153 (5)	−0.0009 (5)	0.0021 (4)	−0.0007 (4)
C3	0.0196 (6)	0.0181 (6)	0.0168 (6)	0.0000 (5)	0.0050 (5)	−0.0007 (5)
C4	0.0163 (6)	0.0147 (6)	0.0181 (6)	0.0004 (4)	−0.0002 (5)	−0.0002 (4)
C5	0.0240 (6)	0.0174 (6)	0.0202 (6)	−0.0003 (5)	0.0072 (5)	0.0024 (5)
C6	0.0246 (6)	0.0155 (6)	0.0224 (6)	0.0011 (5)	0.0055 (5)	−0.0020 (5)

### Geometric parameters (Å, °)

**Table:**

N1—C1	1.3469 (15)	C2—C3	1.3446 (17)
N1—N2	1.3635 (14)	C2—C5	1.4958 (16)
N1—N6	1.4119 (14)	C3—C4	1.4615 (17)
N2—N3	1.2874 (15)	C3—H3	0.953 (15)
N3—N4	1.3745 (14)	C4—C6	1.5018 (16)
N4—C1	1.3207 (15)	C5—H5A	0.9600
N5—C1	1.3623 (15)	C5—H5B	0.9600
N5—C2	1.3820 (15)	C5—H5C	0.9600
N5—H5N	0.920 (18)	C6—H6A	0.9600
N6—C4	1.2897 (16)	C6—H6B	0.9600
			
C1—N1—N2	107.83 (10)	C2—C3—H3	115.8 (9)
C1—N1—N6	137.23 (10)	C4—C3—H3	112.8 (9)
N2—N1—N6	114.45 (9)	N6—C4—C3	132.13 (11)
N3—N2—N1	106.88 (10)	N6—C4—C6	113.82 (10)
N2—N3—N4	110.69 (10)	C3—C4—C6	114.02 (11)
C1—N4—N3	105.82 (10)	C2—C5—H5A	109.5
C1—N5—C2	126.17 (11)	C2—C5—H5B	109.5
C1—N5—H5N	115.3 (10)	H5A—C5—H5B	109.5
C2—N5—H5N	118.4 (10)	C2—C5—H5C	109.5
C4—N6—N1	117.58 (10)	H5A—C5—H5C	109.5
N4—C1—N1	108.78 (10)	H5B—C5—H5C	109.5
N4—C1—N5	122.92 (11)	C4—C6—H6A	109.5
N1—C1—N5	128.29 (11)	C4—C6—H6B	109.5
C3—C2—N5	126.01 (11)	H6A—C6—H6B	109.5
C3—C2—C5	122.01 (11)	C4—C6—H6C	109.5
N5—C2—C5	111.95 (11)	H6A—C6—H6C	109.5
C2—C3—C4	131.03 (12)	H6B—C6—H6C	109.5
			
C1—N1—N2—N3	0.80 (13)	N6—N1—C1—N5	8.2 (2)
N6—N1—N2—N3	174.17 (10)	C2—N5—C1—N4	−174.90 (12)
N1—N2—N3—N4	−0.47 (14)	C2—N5—C1—N1	5.0 (2)
N2—N3—N4—C1	−0.04 (14)	C1—N5—C2—C3	−4.3 (2)
C1—N1—N6—C4	−12.5 (2)	C1—N5—C2—C5	177.67 (12)
N2—N1—N6—C4	176.81 (10)	N5—C2—C3—C4	−7.1 (2)
N3—N4—C1—N1	0.55 (13)	C5—C2—C3—C4	170.75 (12)
N3—N4—C1—N5	−179.54 (11)	N1—N6—C4—C3	0.64 (19)
N2—N1—C1—N4	−0.84 (14)	N1—N6—C4—C6	178.52 (10)
N6—N1—C1—N4	−171.93 (13)	C2—C3—C4—N6	10.9 (2)
N2—N1—C1—N5	179.24 (12)	C2—C3—C4—C6	−167.02 (13)

### Hydrogen-bond geometry (Å, °)

**Table:**

*D*—H···*A*	*D*—H	H···*A*	*D*···*A*	*D*—H···*A*
N5—H5N···N4^i^	0.920 (19)	1.999 (19)	2.9156 (17)	173.5 (15)

Symmetry codes: (i) −*x*+1, −*y*, −*z*+1.

### Table 2 π-π Stacking interactions(Å, °)

**Table:**

Cg_i_	Cg_j_	Cg_i_···Cg_j_	α	Cg_i__perp	Cg_j__perp
Cg_1_	Cg_2_^i^	3.419 (1)	2.83	3.384	3.390

Symmetry code:(i)1+x, y, z; Cg_1_, Cg_2_ are the centroids of the five- and seven-membered rings, respectively. α is the dihedral angle between ring planes and Cg_i__perp is  the perpendicular distance of Cg_i_ on ring *j*, Cg_j__perp is the perpendicular distance of Cg_j_ on ring *i*.
